# Pathophysiology of multiple sclerosis damage and repair: Linking cerebral hypoperfusion to the development of irreversible tissue loss in multiple sclerosis using magnetic resonance imaging

**DOI:** 10.1111/ene.15827

**Published:** 2023-05-30

**Authors:** Daniele Mascali, Alessandro Villani, Antonio M. Chiarelli, Emma Biondetti, Ilona Lipp, Anna Digiovanni, Valeria Pozzilli, Alessandra S. Caporale, Marianna G. Rispoli, Paola Ajdinaj, Maria D'Apolito, Eleonora Grasso, Stefano L. Sensi, Kevin Murphy, Valentina Tomassini, Richard G. Wise

**Affiliations:** ^1^ Department of Neurosciences, Imaging, and Clinical Sciences G. d'Annunzio University of Chieti‐Pescara Chieti Italy; ^2^ Institute for Advanced Biomedical Technologies G. d'Annunzio University of Chieti‐Pescara Chieti Italy; ^3^ Department of Neurophysics Max Planck Institute for Human Cognitive and Brain Sciences Leipzig Germany; ^4^ Cardiff University Brain Research Imaging Centre, School of Psychology Cardiff University Cardiff UK; ^5^ MS Centre, Department of Clinical Neurology SS. Annunziata University Hospital Chieti Italy; ^6^ Department of Paediatrics SS. Annunziata University Hospital Chieti Italy; ^7^ Behavioral Neurology and Molecular Neurology Units, Centre for Advanced Studies and Technology G. d'Annunzio University of Chieti‐Pescara Chieti Italy; ^8^ Cardiff University Brain Research Imaging Centre, School of Physics and Astronomy Cardiff University Cardiff UK

**Keywords:** arterial spin labeling MRI, cerebral blood flow, hypoperfusion, multiple sclerosis, neurodegeneration, white matter lesions

## Abstract

**Background and purpose:**

Reduced cerebral perfusion has been observed in multiple sclerosis (MS) and may contribute to tissue loss both acutely and chronically. Here, we test the hypothesis that hypoperfusion occurs in MS and relates to the presence of irreversible tissue damage.

**Methods:**

In 91 patients with relapsing MS and 26 healthy controls (HC), gray matter (GM) cerebral blood flow (CBF) was assessed using pulsed arterial spin labeling. GM volume, T1 hypointense and T2 hyperintense lesion volumes (T1LV and T2LV, respectively), and the proportion of T2‐hyperintense lesion volume that appears hypointense on T1‐weighted magnetic resonance imaging (T1LV/T2LV) were quantified. GM CBF and GM volume were evaluated globally, as well as regionally, using an atlas‐based approach.

**Results:**

Global GM CBF was lower in patients (56.9 ± 12.3 mL/100 g/min) than in HC (67.7 ± 10.0 mL/100 g/min; *p* < 0.001), a difference that was widespread across brain regions. Although total GM volume was comparable between groups, significant reductions were observed in a subset of subcortical structures. GM CBF negatively correlated with T1LV (*r* = −0.43, *p* = 0.0002) and T1LV/T2LV (*r* = −0.37, *p* = 0.0004), but not with T2LV.

**Conclusions:**

GM hypoperfusion occurs in MS and is associated with irreversible white matter damage, thus suggesting that cerebral hypoperfusion may actively contribute and possibly precede neurodegeneration by hampering tissue repair abilities in MS.

## INTRODUCTION

Cerebral hypoperfusion, as quantified by perfusion‐weighted magnetic resonance imaging (MRI) [1], has been demonstrated to be present in MS [2, 3, 4, 5] from the earliest phases of the disease, both acutely, preceding lesion formation, and chronically, preceding cerebral atrophy [6, 7, 8]. Hyperproduction of vasoconstrictor and of inflammatory response mediators such as endothelin‐1 has been proposed as a cause of reduced cerebral blood supply [9]. Hypoperfusion may play a role in the development of brain damage in multiple sclerosis (MS), as suggested by an increase of T1‐hypointense lesion volume (T1LV) in regions with reduced cerebral blood flow (CBF) [10, 11, 12], a result that hints at the possibility of a causal relationship between hypoperfusion and tissue loss.

The volume of hyperintense lesions on T2‐weighted MRI (T2LV) can be considered a nonspecific marker of brain inflammation and its consequences. Hyperintense lesions on T2‐weighted MRI can remain stable, expand, shrink, or rarely, disappear over time [13, 14]. Moreover, hyperintense lesions on T2‐weighted MRI are weakly related to clinical disability [15] and their development can be limited by disease‐modifying therapy [16, 17]. Conversely, hypointense lesions on T1‐weighted MRI are chronic in nature, accumulating over time, and are more strongly associated with clinical disability [18, 19]. T1LV can be regarded as representative of irreversible brain damage. The T1LV to T2LV ratio (T1LV/T2LV) is a potential alternative marker of irreversible tissue loss that may index a reduced tissue repair ability, namely the fraction of global lesion volume that results in tissue loss [2, 14, 20, 21, 22].

Here, we test the hypothesis that the presence of irreversible tissue damage is greater in patients showing lower cerebral perfusion. First, we quantify gray matter (GM) perfusion in MS and healthy controls (HC) using pulsed arterial spin labeling (ASL). Second, we measure its association with the development of irreversible white matter (WM) damage, in terms of both absolute amount of irreversible lesional damage and proportion of irreversible lesional damage of the global lesional damage (T1LV/T2LV). We expect that a reduction of GM perfusion occurs in MS and is more pronounced in patients with irreversible tissue damage, thus suggesting that patients with greater hypoperfusion are at higher risk of tissue loss.

## MATERIALS AND METHODS

### Participants and clinical assessment

Right‐handed patients with a diagnosis of relapsing–remitting MS [23] were included in the study if they were between 18 and 60 years of age, had no relapse or change in treatment in the 3 months before study entry, and did not suffer from any other neurological or psychiatric conditions. The neurological examination included the Expanded Disability Status Scale (EDSS). Sex‐matched HC were also recruited. The study was approved by the National Health Service South‐West Ethic Committee and the Cardiff University Health Board Research and Development Committee. All participants provided written informed consent. Data from the described cohort has been published previously [24, 25].

### MRI acquisitions

Magnetic resonance images were acquired on a 3‐T Signa HDx MRI system (GE Medical Systems) equipped with an eight‐channel receive‐only head radiofrequency coil. Structural images, acquired for the purpose of lesion identification, image registration, and volumetric quantification, included a T1‐weighted three‐dimensional (3D) fast spoiled gradient‐recalled‐echo acquisition (slice orientation = sagittal, resolution = 1 × 1 × 1 mm^3^, echo time [TE] = 3.0 ms, repetition time [TR] = 7.8 ms, inversion time [TI] = 450 ms, flip angle [FA] = 20°, total acquisition time = 7.5 min), a 2D proton density (PD)/T2‐weighted dual‐echo sequence (slice orientation = axial, resolution = 0.94 × 0.94 × 3 mm^3^, slice gap = 1.5 mm, TE = 9.0/80.6 ms, TR = 3000 ms, FA = 90°, total acquisition time = 2 min), and a T2 2D fluid‐attenuated inversion recovery (FLAIR) sequence (slice orientation = axial, resolution = 0.86 × 0.86 × 3 mm^3^, slice gap = 1.5 mm, TE = 122.3 ms, TR = 9502 ms, TI = 2250 ms, FA = 90°, total acquisition time = 3 min). ASL data were collected with a multi‐inversion time 2D PICORE QUIPSS II sequence with two spiral k‐space readouts at a short and a long TE (slice orientation = axial, resolution 3 × 3 × 7 mm^3^, slice gap = 1 mm, TE_1_ = 3 ms, TE_2_ = 29 ms, TR = 4000 ms, total acquisition time = 5 min), and with the saturation pulse for temporally defined bolus set at 700 ms. Four inversion times were used (TI = 1100, 1400, 1700, and 2000 ms), each collecting eight signal averages to increase the signal‐to‐noise ratio. An image with the same readout as the ASL data and effectively infinite TR was acquired to estimate the equilibrium magnetization of water for calibration purposes. To correct for the coil sensitivity profile, a minimum contrast image was collected (TE = 11 ms, TR = 2000 ms). Image analysis was performed using FSL [26] and in‐house code running on MATLAB 2020b (MathWorks).

#### Lesion identification and tissue segmentation

T2‐hyperintense lesions were semiautomatically contoured on the T2 FLAIR image using Jim (v6, Xinapse) by an operator (I.L.) experienced in producing T2‐hyperintense lesion masks. Then, the T2 FLAIR image was aligned to the structural T1‐weighted image using FSL FLIRT [27, 28] with a 6‐*df*, rigid‐body transformation, and the resulting transformation matrix was applied to the T2‐hyperintense lesion mask. T1‐hypointense voxels were identified as voxels within the registered T2‐hyperintense lesion mask with a T1‐weighted intensity equal to or lower than the average T1‐weighted intensity of the GM (defined as partial volume estimate [PVE] > 0.5) [19, 25]. The total volumes of the T2‐hyperintense and T1‐hypointense lesions (T2LV and T1LV) were assessed using the masks in the T1‐weighted image space. The ratio between T1LV and T2LV (T1LV/T2LV) was calculated as a measure of irreversible WM damage complementary to T1LV.

For GM and WM segmentation, T1‐weighted images were lesion‐filled using the FSL lesion_filling tool [29]. T1‐weighted images from HC and lesion‐filled T1‐weighted images from the MS patients were segmented using FSL FAST [30] to obtain PVEs of tissue compartments. As FAST systematically underestimated the volume of subcortical structures, FSL FIRST [31] was also applied to improve segmentation in these regions. Then, subcortical structures were removed from the GM PVE estimate provided by FAST and replaced with those segmented by FIRST. For regional group analysis, linear (FLIRT) and nonlinear (FNIRT) [26] registrations were calculated and combined into a single transformation that mapped native‐space structural images to the standard Montreal Neurological Institute (MNI) space. GM volume, normalized for subject head size, was estimated using FSL SIENAX on the FAST and FIRST combined output images [32, 33].

#### Perfusion quantification

ASL control and tag images at the short TE were motion corrected using the PD image as the reference volume (MCFLIRT) [27] and brain extracted (BET) [32]. Pairwise subtraction of control and tag images was performed, yielding a perfusion‐weighted image at each TI, replicated for the eight repetitions. Before averaging across repetitions, outlier volumes were discarded following a dedicated filtering procedure [34], which was applied to each TI separately. Across all subjects, the number of discarded repetitions ranged from 0 to 1 for TI = 1100, 1400, and 1700 ms, and from 0 to 2 for TI = 2000 ms; on average, 0.65 ± 0.01 volumes per subject were discarded.

The cleaned and averaged series of perfusion‐weighted images were fed into oxford_asl (BASIL) [35] for CBF quantification. Oxford_asl was run with spatial regularization, estimation of the macrovascular component, coil profile sensitivity correction, and perfusion calibration using the cerebrospinal fluid signal extracted from the PD‐weighted volume. For the sensitivity correction, the bias field was used, which was estimated on the minimum contrast image via the SPM12 segmentation routine [36]. The resulting CBF maps were transformed into MNI space. The spatial transformation to standard space was obtained by concatenating a linear transformation (FLIRT) from the PD‐weighted to the T1‐weighted image, and the previously derived nonlinear transformation from the structural space to the MNI space. Consistently with consensus guidelines for ASL [37], CBF values were only evaluated in GM regions.

#### Extraction of regional CBF and GM volumes

Both GM volume and GM CBF were evaluated at the regional level using the AAL2 atlas [38], which comprises 120 regions of interests (ROIs) from both cortical and subcortical structures. The GM volume of each ROI was extracted from the GM PVE in native space after transforming the AAL2 atlas to the high‐resolution subject‐specific space. To normalize volumes for head size differences, the within‐ROI summed GM PVE was scaled by the SIENAX factor. Regional values of CBF were calculated in MNI space by taking the median CBF within each ROI. To mitigate any potential impact of tissue loss on the regional CBF calculation, only voxels with a GM PVE of at least 50% were considered.

### Statistical analysis

Demographic and clinical characteristics were compared between groups using two‐tailed Welch *t*‐tests, except for sex differences, which were assessed using a chi‐squared test. Between‐group differences in CBF and GM volumes were assessed via two‐tailed Welch *t*‐tests. Because age and education were significantly different between the groups considered, the tests were performed after regressing (covarying) out the effect of age and education. Associations between CBF and lesion volumes (T1LV, T2LV, and T1LV/T2LV) were tested via Spearman correlations. Correlations were calculated after removing any data point more than three scaled median absolute deviations away from the median, and regressing out the effects of age, education, and regional GM volume. When performing regionwise analysis, correction for multiple comparisons was carried out using the false discovery rate (FDR) at *p*FDR < 0.05.

## RESULTS

### Demographic and clinical characteristics

Of 104 MS patients for whom ASL data were available, 13 were excluded due to poor ASL data quality (e.g., excess motion) or problems with image acquisition and reconstruction, leaving 91 patients included in the analyses. None of the 26 HC was discarded following data quality checks.

Table 1 reports the demographic and clinical characteristics of the participants. MS patients were on average older (*p* = 0.026) and had shorter education than HC (*p* < 0.001).

**TABLE 1:** Demographic, clinical, and magnetic resonance imaging characteristics.

Characteristic	MS, *n* = 91	HC, *n* = 26	*p*
Age, years	43.5 ± 9.8	38.5 ± 11.0	0.026
Sex, M/F	26/65	11/15	0.184
Education, years	15.5 ± 3.7	19.8 ± 4.3	<0.001
Disease duration, years	12.0 ± 7.9	–	–
EDSS score	2.6 ± 1.5	–	–
Patients on DMTs, *n*	31	–	–
GM volume, mL	793 ± 53	799 ± 32	0.378
WM volume, mL	741 ± 36	752 ± 37	0.15
T2‐hyperintense LV, mm^3^	17.7 ± 17.3	–	–
T1‐hypointense LV, mm^3^	1.8 ± 2.2	–	–

*Note:* Data are presented as mean ± SD.

Abbreviations: DMT, disease‐modifying therapy; EDSS, Expanded Disability Status Scale; F, female; GM, gray matter; HC, healthy controls; LV, lesion volume; M, male; MS, multiple sclerosis; WM, white matter.

### GM perfusion and volumetry

**FIGURE 1:**
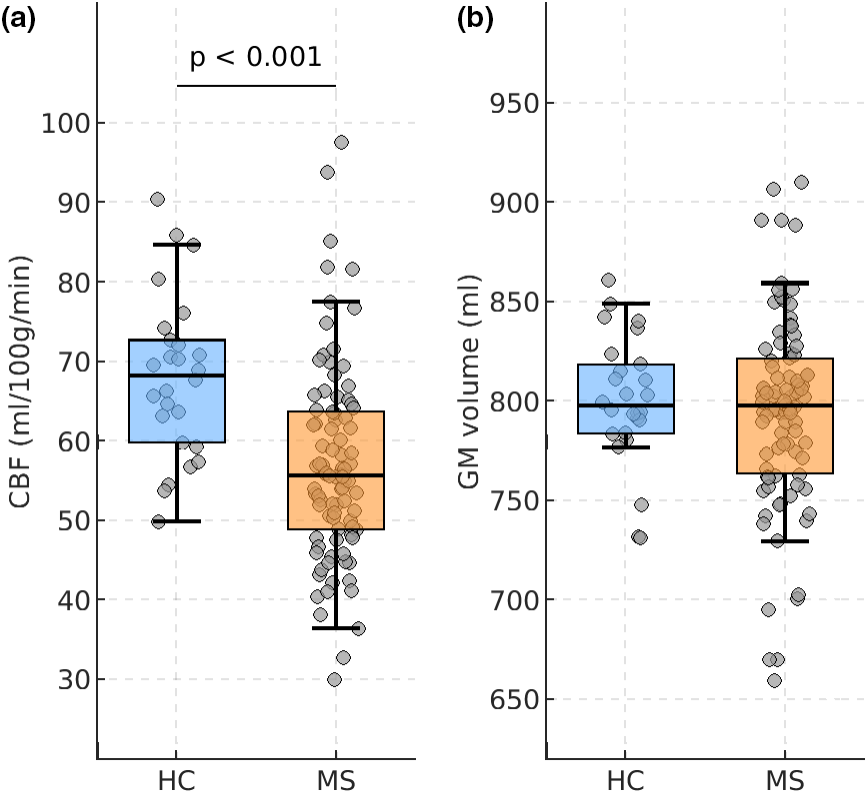
Global gray matter (GM) cerebral blood flow (CBF) and GM volume in multiple sclerosis (MS) patients and in healthy controls (HC). The distributions of GM CBF (a) and GM volume normalized for head size (b) are shown for HC (blue) and for MS patients (orange). Mean ± SD GM volume was similar between groups. Box plots show the median and the 25th/75th percentiles (edges of the boxes); the whiskers extend to the closest data point within 1.5 times the interquartile range. Probability values are shown, when significant. [Colour figure can be viewed at wileyonlinelibrary.com]

Figure 1 shows whole‐brain GM CBF and GM volume in patients compared to HC, controlling for the effects of age and education. Patients had lower CBF compared to HC (MS: 56.9 ± 12.3 mL/100 g/min; HC: 67.7 ± 10.0 mL/100 g/min; *p* < 0.001), whereas global GM volumes were statistically comparable (MS: 793 ± 53 mL; HC: 799 ± 32 mL; *p* = 0.378).

**FIGURE 2:**
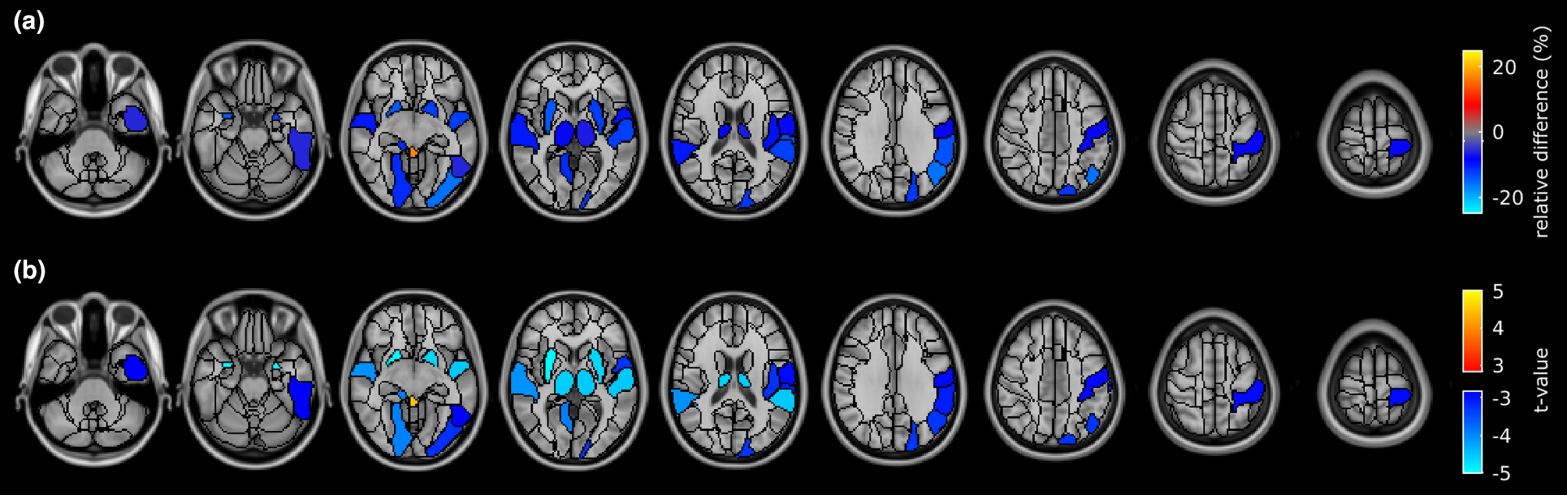
Regional differences in gray matter (GM) volume between healthy controls (HC) and multiple sclerosis patients. (a) Between‐group difference in GM volume compared to HC. (b) Welch test *t*‐map. Both maps are thresholded at false discovery rate‐adjusted *p* < 0.05. Images are displayed in the radiological convention (the left hemisphere is on the right side of the image). [Colour figure can be viewed at wileyonlinelibrary.com]

Figure 2 shows the regionwise analysis comparing GM volume of MS patients with that of HC. In patients, between‐group differences in regional GM volume were found (*p*FDR < 0.05), which were more localized than the widespread hypoperfusion. In patients, regional brain atrophy was primarily found in subcortical structures such as thalamus, putamen, and amygdala. However, involvement of cortical structures was also observed in the bilateral superior temporal lobe, as well as in regions of the left frontal–occipital–parietal cortex (i.e., superior and inferior occipital gyrus, supramarginal and angular gyrus, postcentral gyrus, and pars opercularis of the inferior frontal gyrus). In regions with significant between‐group GM volume differences, the difference in GM volumes between HC and MS patients ranged from −15.8% to −5.7% (Table S1), with a mean ± SD of −11.3 ± 3.2%.

**FIGURE 3:**
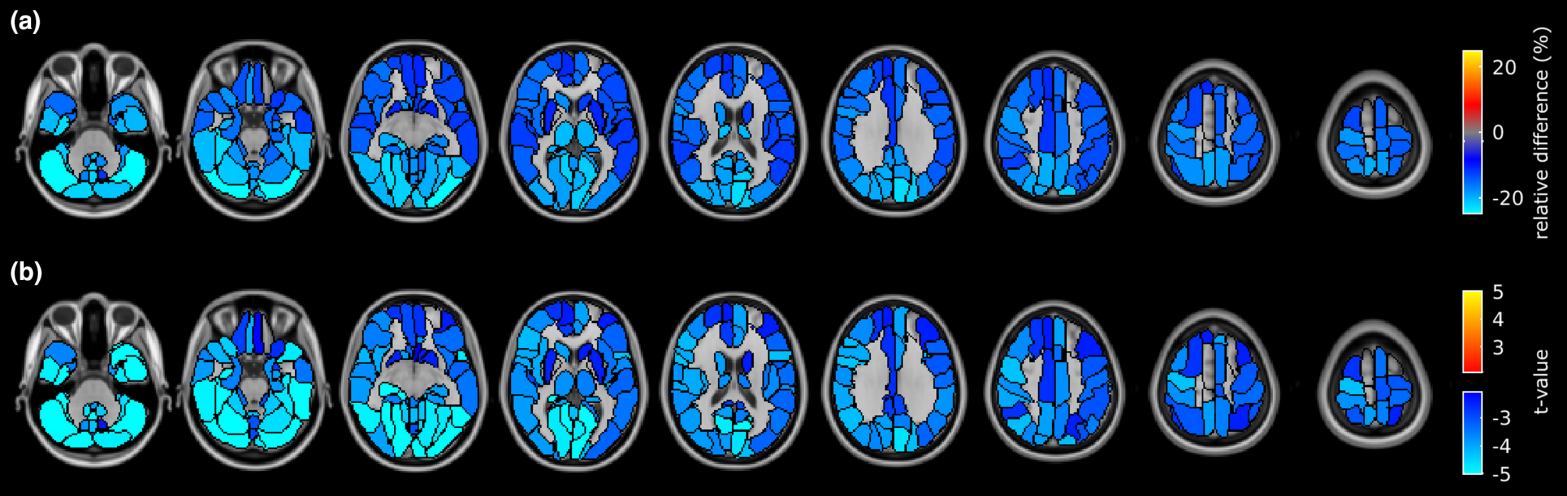
Regional differences in gray matter cerebral blood flow between healthy controls (HC) and multiple sclerosis patients. Brain regions were classified using the AAL2 atlas and represented using a colour map. (a) Between‐group difference in perfusion compared to HC. (b) Welch test *t*‐map. Both maps are thresholded at false discovery rate‐adjusted *p* < 0.05. Images are displayed in the radiological convention. [Colour figure can be viewed at wileyonlinelibrary.com]

Figure 3 shows the regionwise analysis comparing GM CBF of MS patients with that of HC. Significant hypoperfusion (*p*FDR < 0.05) was identified throughout cortical and subcortical regions. The relative significant difference in CBF between HC and patients ranged from −32.0% to −10.5% (Table S1), with an average of −16.8% ± 3.8%. The spatial pattern of statistical differences in CBF between patients and HC was unchanged when including the regionwise GM volume as a covariate of no interest.

### GM perfusion and WM lesions

**FIGURE 4:**
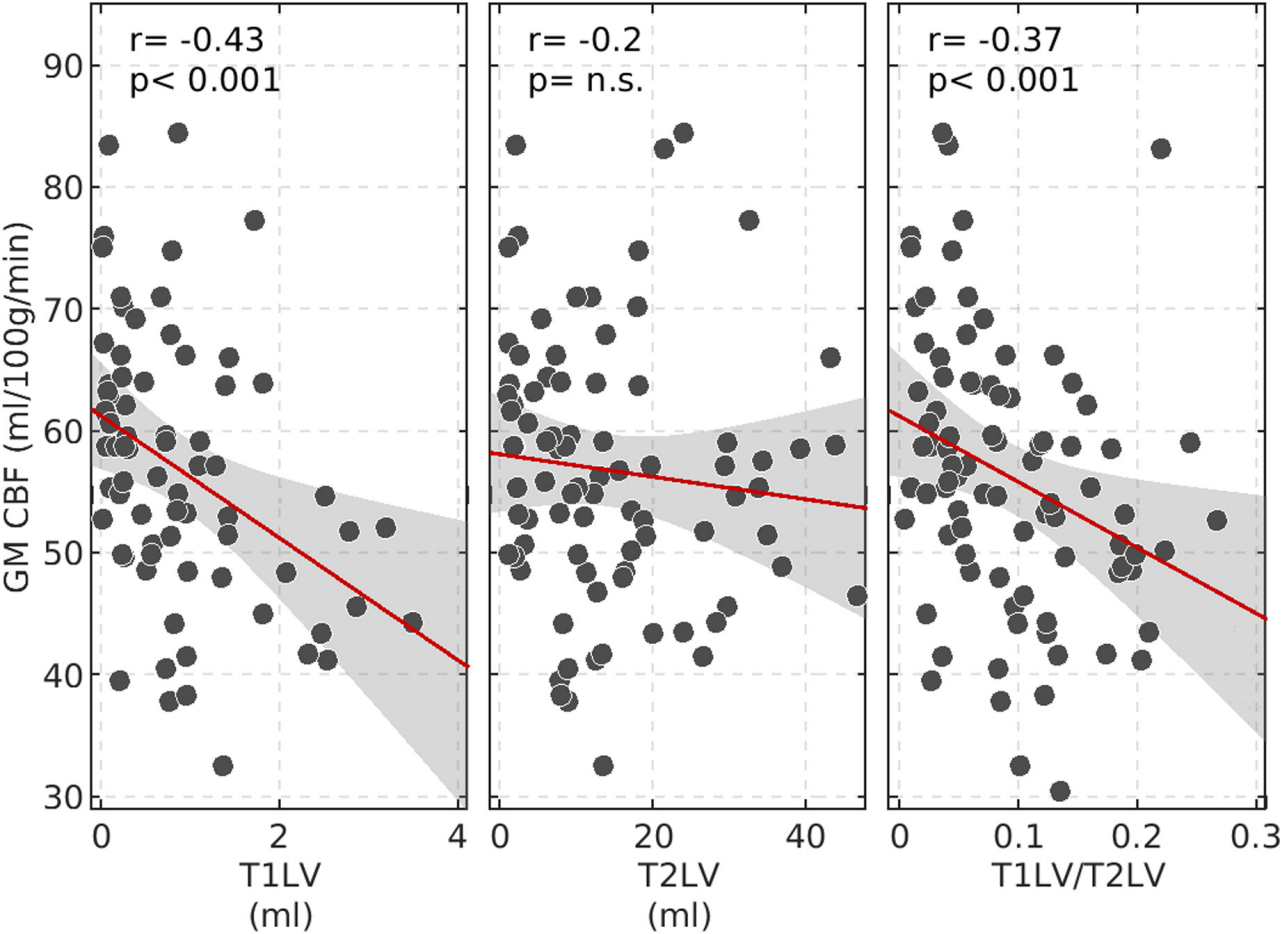
Correlations between gray matter (GM) cerebral blood flow (CBF) and lesion volumes and between T1‐hypointense lesion volume (T1LV) and demographic, clinical, and radiological characteristics. Correlation between global GM CBF and T1LV, T2‐hyperintense lesion volume (T2LV), and T1LV/T2LV shows a significant association between GM CBF and T1LV and T1LV/T2LV. n.s., not significant. [Colour figure can be viewed at wileyonlinelibrary.com]

**FIGURE 5:**
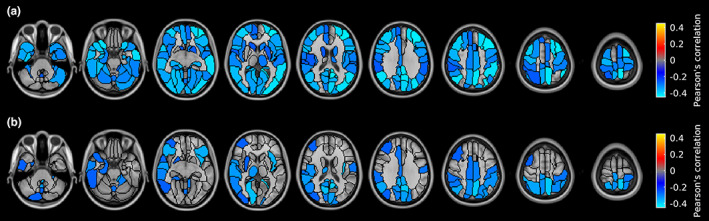
Regionwise correlations of gray matter (GM) cerebral blood flow (CBF) with T1‐hypointense lesion volume (T1LV) and T1LV/T2‐hypointense lesion volume (T2LV) in multiple sclerosis patients. (a) Correlation between GM CBF and T1LV. (b) Correlation between GM CBF and T1LV/T2LV. The correlation map is thresholded at false discovery rate‐adjusted *p* < 0.05. Images are displayed in the radiological convention. [Colour figure can be viewed at wileyonlinelibrary.com]

Figure 4 shows the associations between global GM CBF and the three metrics derived from WM lesion volumes (T1LV, T2LV, and T1LV/T2LV). Whereas no significant correlation was found between GM CBF and T2LV, negative correlation was found between GM CBF and T1LV (*r* = −0.43, *p* < 0.001) and between GM CBF and T1LV/T2LV (*r* = −0.37, *p* < 0.001). T1LV and T1LV/T2LV were not associated with potential confounders such as age, disease duration, or GM volume. Figure 5 shows localized associations between GM CBF and T1LV (Figure 5a) and between GM CBF and T1LV/T2LV (Figure 5b). A widespread correlation (*p*FDR < 0.05) was observed between GM CBF and both T1LV and T1LV/T2LV, ranging from *r* = −0.25 to *r* = −0.43 with a mean ± SD of *r* = −0.33 ± 0.05, and from *r* = −0.39 to *r* = 0.04 with a mean ± SD of *r* = −0.22 ± 0.09, respectively, across significant regions. No region exhibited a positive association of GM CBF with T1LV and T1LV/T2LV. A summary of the main findings for each ROI in the AAL2 atlas is reported in Table S1.

## DISCUSSION

This study investigates alteration in GM perfusion and its association with irreversible damage in MS. GM hypoperfusion was observed in patients compared to HC, with more pronounced CBF reductions in patients who were prone to or presented with higher irreversible WM damage. This relationship was specific to the presence of irreversible tissue damage, characterized by T1‐hypointense lesions, rather than the general accumulation of MRI burden of disease or aging. A negative correlation, widespread across brain regions, was found between GM CBF and T1LV, as well as between GM CBF and T1LV/T2LV, supporting the hypothesis of hypoperfusion consequent to inflammation as a contributor to irreversible tissue loss in MS.

### GM volumetry and hypoperfusion

Although localized GM volume loss in patients was observed in brain areas known to be affected by early atrophy in MS [39, 40], no significant global reduction in GM volume was found in patients compared to HC. This finding could be explained by the relatively small number of GM regions affected by significant atrophy in patients, which failed to result in a significant global atrophy across the whole GM volume.

Global GM CBF in HC was consistent with that measured in previous studies using MRI approaches such as ASL [12] and dynamic susceptibility contrast (DSC) [11] or positron emission tomography [41]. Mean GM CBF in MS patients, instead, was reduced when compared to HC, with a widespread involvement of several brain regions [6]. This widespread GM hypoperfusion in the presence of focal GM atrophy suggests that (i) perfusion changes in the patients are not wholly dependent on changes in tissue volume and (ii) hypoperfusion and tissue loss may develop over different timescales, with hypoperfusion possibly preceding detectable tissue damage on MRI [9]. This hypothesis of “primary ischemia” is corroborated by our finding of lack of a direct relationship between T1LV and GM volume and is independently supported by diffusion‐based and DSC MRI‐based studies showing that, in the normal‐appearing corpus callosum, CBF correlates positively with mean diffusivity, but not with fractional anisotropy, a result more consistent with the *“*primary ischemia” hypothesis than with hypoperfusion secondary to axonal degeneration [42, 43]. However, the lack of correspondence between GM areas with the most marked CBF reduction and those with the largest atrophy suggests that primary ischemia is not the only contributor to the development of neurodegeneration, at least within the extent of damage represented in our cohort.

### GM hypoperfusion and irreversible WM damage

GM CBF showed a specific, significant negative correlation with T1LV and with T1LV/T2LV. Both these correlations indicated an association between hypoperfusion and irreversible WM damage or lack of repair, supporting the hypothesis that GM hypoperfusion, reflecting a global energetic tissue dysfunction [4, 7], is coupled to the severity of WM damage and potentially suggesting an impairment of repair abilities in the brain with reduced perfusion, although causality remains to be established. Hypoperfusion may create an environment in which repair mechanisms, such as remyelination, are impaired [2, 12]. In this condition, metabolic failure, which is characterized by mitochondrial dysfunction and “virtual hypoxia” [44, 45], could determine the accumulation of oxygen radicals and the depletion of energetic resources necessary to preserve and repair lesions, establishing a vicious circle leading to permanent damage. Such damage may manifest as irreversible lesions in the WM and a tendency toward neurodegeneration in the GM [2, 7, 46]. The association between GM hypoperfusion and T1LV or T1LV/T2LV was spatially widespread. This suggests that the tendency for focal WM tissue loss, or lack of repair, may be linked to global factors affecting perfusion that, in turn, may depend on individual disease traits or stage of disease rather than a localized effect of inflammation.

### Limitations

This study presents some limitations. First, the cross‐sectional nature of this study permits only the testing of associations between perfusion and measures of irreversible damage at one point in time along the disease course, but longitudinal studies would be necessary to establish the causal link between hypoperfusion and the development of tissue damage. Second, the absence of contrast administration in the study did not allow us to identify enhancing lesions and thus exclude them from the T1‐hypointense lesion volume calculation. It may be, therefore, that a small proportion of the T1‐hypointense lesions were not purely chronic injuries with irreversible tissue destruction. On the other hand, only patients with no relapsing in the 3 months preceding recruitment were included, which makes the presence of enhancing lesions less likely. Third, some limitations of the present study were inherent to the ASL technique.

The CBF estimate depends on the ASL efficiency, the variability of which as a function of the afferent main brain artery may create some spurious changes in the estimated regional perfusion, for example, when spatially comparing brain regions irrorated by the anterior and posterior circulations [47]. Nonetheless, this effect should, on average, equally affect each group of patients, without altering the estimated between‐groups perfusion changes, but only the statistical relevance of the findings due to noise variability. Of note, average CBF maps did not show any spatial characteristics suggesting a possible effect of labeling efficiency on the regional findings of the work. In addition, ASL offers low sensitivity as a measure of CBF in the WM due to the long arrival time of blood in this compartment, particularly in MS patients [48]. Hence, our data did not enable us to quantify WM CBF and compare it with GM CBF or lesion load, although GM and WM hypoperfusion have been previously observed to be coupled from the early stages of MS [8]. Furthermore, our data could not inform on a potential periventricular gradient of hypoperfusion, which future studies could assess in relation with the known periventricular gradient of MS lesion occurrence [49].

## CONCLUSIONS

This cross‐sectional study demonstrates an association between GM hypoperfusion and irreversible WM damage. Despite the need for further longitudinal assessments, hypoperfusion may be associated with impaired tissue repair via the alteration in nutrient supply, which may contribute to, and possibly precede, tissue loss in MS. Cerebral perfusion through ASL can contribute to improving our understanding of the pathophysiology of MS damage and repair, and it may be able to identify patients at risk of irreversible tissue damage.

## AUTHOR CONTRIBUTIONS

**Richard G. Wise:** Conceptualization; funding acquisition; supervision; methodology. **Daniele Mascali:** Methodology; formal analysis; writing – original draft; writing – review and editing. **Alessandro Villani:** Conceptualization; writing – original draft; writing – original draft. **Antonio M. Chiarelli:** Conceptualization; writing – original draft; methodology. **Emma Biondetti:** Methodology; writing – original draft. **Ilona Lipp:** Methodology; data curation. **Anna Digiovanni:** Visualization. **Valeria Pozzilli:** Visualization. **Alessandra S. Caporale:** Visualization, writing – review and editing. **Marianna Rispoli:** Visualization. **Paola Ajdinaj:** Visualization. **Maria D’Apolito:** Visualization. **Eleonora Grasso:** Visualization. **Stefano L. Sensi:** Visualization; supervision. **Kevin Murphy:** Visualization; data curation; methodology. **Valentina Tomassini:** Conceptualization; funding acquisition; supervision; writing – original draft.

## FUNDING INFORMATION

The study was funded by the MS Society UK and, in part, by the Wellcome Trust (WT200804). The authors have applied a CC BY public copyright licence to any author‐accepted manuscript version arising from this submission.

## CONFLICT OF INTEREST STATEMENT

V.T. has received honoraria, research support, and travel grants from Alexion, Biogen, Merck Serono, Roche, Novartis, Almirall, Sanofi, Viatris, Janssen and Bristol Myers Squibb. The other authors have no competing interests to declare.

## Supporting information


TABLE S1

## Data Availability

Data for the present study are available upon reasonable request.
