# Supplementary material for: Pathophysiology of multiple sclerosis damage and repair: Linking cerebral hypoperfusion to the development of irreversible tissue loss in multiple sclerosis using magnetic resonance imaging
Source: Eur J Neurol. 2023 May 30;30(8):2348–56. doi: 10.1111/ene.15827 (PMC7615142; doi:10.1111/ene.15827)
Supplement: Supplementary file 1 — TABLE S1 [file ENE-30-2348-s001.docx]

**Mascali et al. Supplementary Material**

**Supplementary Table S1**. Region-wise CBF values and GM volumes. For each AAL2 region, the table reports the between-group difference in CBF and GM volumes (as the relative difference and *t*-value) and the Pearson’s correlation between CBF and T1LV and between CBF and T1LV/T2LV. Significant values (*p*FDR < 0.05) are shown in bold.

|  |  | **CBF**  **MS > HC** | | **GM Volume**  **MS > HC** | | **CBF vs**  **T1LV** | **CBF vs**  **T1LV/T2LV** |
| --- | --- | --- | --- | --- | --- | --- | --- |
| **N** | **Brain Region** | **Relative Difference (%)** | ***t*-value** | **Relative Difference (%)** | ***t*-value** | **r** | **r** |
| 1 | Left Precentral gyrus | **-14.8** | **-3.1** | -5.1 | -2.0 | **-0.32** | -0.22 |
| 2 | Right Precentral gyrus | **-18.1** | **-4.3** | -3.3 | -1.0 | **-0.36** | -0.21 |
| 3 | Left Superior Frontal gyrus | -9.4 | -1.6 | 4.7 | 1.7 | **-0.40** | -0.13 |
| 4 | Right Superior Frontal gyrus | **-12.7** | **-2.7** | 1.0 | 0.4 | **-0.32** | -0.25 |
| 5 | Left Middle Frontal gyrus | **-13.0** | **-2.4** | 3.1 | 1.1 | **-0.43** | -0.22 |
| 6 | Right Middle Frontal gyrus | **-15.7** | **-3.7** | 3.0 | 1.3 | **-0.39** | **-0.26** |
| 7 | Left Frontal Operculum | **-14.8** | **-4.3** | -3.0 | -0.8 | **-0.36** | -0.17 |
| 8 | Right Frontal Operculum | **-15.7** | **-4.0** | 1.7 | 0.5 | **-0.35** | -0.18 |
| 9 | Left Inferior Frontal gyrus, Triangular part | **-13.0** | **-3.2** | -0.7 | -0.2 | **-0.34** | -0.18 |
| 10 | Right Inferior Frontal gyrus, Triangular part | **-15.2** | **-4.2** | -0.2 | -0.1 | **-0.32** | -0.24 |
| 11 | Left Inferior Frontal gyrus, Orbital part | **-13.2** | **-3.3** | -5.3 | -1.4 | **-0.36** | **-0.35** |
| 12 | Right Inferior Frontal gyrus, Orbital part | **-14.6** | **-3.5** | -4.5 | -1.4 | **-0.31** | **-0.36** |
| 13 | Left Inferior Frontal gyrus, Opercular part | **-12.6** | **-3.7** | **-9.5** | **-3.3** | **-0.31** | -0.19 |
| 14 | Right Inferior Frontal gyrus, Opercular part | **-14.9** | **-4.0** | -4.1 | -1.8 | **-0.25** | **-0.32** |
| 15 | Left Supplementary Motor area | **-14.2** | **-3.4** | 6.5 | 1.4 | **-0.28** | -0.15 |
| 16 | Right Supplementary Motor area | -9.9 | -1.9 | -0.6 | -0.1 | -0.23 | -0.18 |
| 17 | Left Olfactory cortex | **-11.9** | **-2.1** | -5.5 | -2.5 | 0.05 | 0.00 |
| 18 | Right Olfactory cortex | **-16.3** | **-3.2** | -4.7 | -2.2 | -0.06 | -0.10 |
| 19 | Left Superior Frontal gyrus, Medial | **-15.4** | **-4.0** | -2.8 | -0.9 | -0.22 | -0.13 |
| 20 | Right Superior Frontal gyrus, Medial | **-10.9** | **-2.4** | -2.8 | -0.9 | **-0.26** | -0.17 |
| 21 | Left Superior Frontal gyrus, Medial Orbital | **-10.5** | **-2.6** | -0.2 | -0.1 | **-0.27** | -0.07 |
| 22 | Right Superior Frontal gyrus, Medial Orbital | **-11.7** | **-2.9** | 0.2 | 0.1 | **-0.34** | -0.26 |
| 23 | Left Rectus gyrus | **-12.5** | **-2.2** | 3.8 | 1.1 | -0.02 | 0.03 |
| 24 | Right Rectus gyrus | **-18.5** | **-4.1** | 5.3 | 1.7 | -0.15 | -0.04 |
| 25 | Left Medial Orbital gyrus | -2.7 | -0.4 | 4.8 | 1.6 | -0.12 | 0.04 |
| 26 | Right Medial Orbital gyrus | **-13.7** | **-2.8** | 3.8 | 1.5 | -0.12 | -0.05 |
| 27 | Left Anterior Orbital gyrus | -11.6 | -1.8 | 6.8 | 2.1 | **-0.26** | -0.05 |
| 28 | Right Anterior Orbital gyrus | **-20.6** | **-4.2** | 5.6 | 2.0 | **-0.40** | -0.19 |
| 29 | Left Posterior Orbital gyrus | **-12.0** | **-2.6** | 1.0 | 0.4 | **-0.38** | -0.12 |
| 30 | Right Posterior Orbital gyrus | **-18.0** | **-4.5** | 3.7 | 1.2 | **-0.41** | -0.06 |
| 31 | Left Lateral Orbital gyrus | **-17.8** | **-4.2** | 1.0 | 0.3 | -0.20 | -0.14 |
| 32 | Right Lateral Orbital gyrus | **-18.7** | **-3.3** | -5.2 | -1.2 | **-0.26** | -0.21 |
| 33 | Left Insular cortex | **-13.4** | **-3.4** | -5.0 | -2.5 | **-0.32** | **-0.35** |
| 34 | Right Insular cortex | **-15.5** | **-4.0** | -6.0 | -2.7 | **-0.29** | -0.24 |
| 35 | Left Cingulate Anterior gyrus | **-15.7** | **-3.8** | 2.1 | 0.6 | -0.19 | -0.09 |
| 36 | Right Cingulate Anterior gyrus | **-12.3** | **-2.8** | 3.8 | 1.1 | **-0.28** | -0.14 |
| 37 | Left Middle Cingulate gyrus | **-15.6** | **-3.7** | -2.6 | -1.0 | **-0.34** | **-0.30** |
| 38 | Right Middle Cingulate gyrus | **-11.5** | **-2.6** | -1.2 | -0.6 | **-0.30** | **-0.28** |
| 39 | Left Posterior Cingulate gyrus | **-18.1** | **-2.9** | -9.5 | -2.4 | **-0.41** | **-0.34** |
| 40 | Right Posterior Cingulate gyrus | **-17.9** | **-2.8** | -6.2 | -1.7 | **-0.35** | **-0.35** |
| 41 | Left Hippocampus | **-17.0** | **-3.9** | 1.8 | 0.9 | **-0.35** | -0.25 |
| 42 | Right Hippocampus | **-19.8** | **-5.3** | 4.7 | 2.2 | **-0.35** | -0.23 |
| 43 | Left Parahippocampal gyrus | **-15.1** | **-3.8** | 3.6 | 1.7 | -0.17 | -0.22 |
| 44 | Right Parahippocampal gyrus | **-15.7** | **-3.5** | 1.5 | 0.7 | **-0.30** | **-0.27** |
| 45 | Left Amygdala | **-12.8** | **-2.7** | **-12.8** | **-5.0** | -0.24 | -0.23 |
| 46 | Right Amygdala | -10.9 | -1.7 | **-14.2** | **-5.1** | **-0.39** | -0.13 |
| 47 | Left Calcarine fissure and surrounding cortex | **-21.6** | **-5.0** | -4.7 | -1.4 | **-0.35** | -0.25 |
| 48 | Right Calcarine fissure and surrounding cortex | **-18.8** | **-4.8** | -8.3 | -2.0 | **-0.34** | **-0.37** |
| 49 | Left Cuneus | **-22.7** | **-4.7** | -5.1 | -1.2 | **-0.40** | **-0.33** |
| 50 | Right Cuneus | **-19.5** | **-3.9** | -11.4 | -2.7 | **-0.30** | **-0.29** |
| 51 | Left Lingual gyrus | **-19.8** | **-4.8** | -7.0 | -2.7 | **-0.32** | -0.26 |
| 52 | Right Lingual gyrus | **-22.0** | **-5.7** | **-11.0** | **-3.9** | **-0.36** | -0.24 |
| 53 | Left Superior Occipital gyrus | **-21.8** | **-4.3** | **-12.0** | **-3.3** | **-0.34** | **-0.31** |
| 54 | Right Superior Occipital gyrus | **-19.3** | **-3.6** | -10.0 | -2.3 | -0.20 | -0.21 |
| 55 | Left Middle Occipital gyrus | **-17.5** | **-3.2** | -4.3 | -1.0 | **-0.42** | -0.25 |
| 56 | Right Middle Occipital gyrus | **-19.2** | **-3.9** | -6.4 | -1.4 | **-0.36** | **-0.26** |
| 57 | Left Inferior Occipital gyrus | **-25.3** | **-5.3** | **-15.7** | **-3.2** | **-0.40** | -0.19 |
| 58 | Right Inferior Occipital gyrus | **-20.1** | **-4.0** | -5.3 | -0.9 | **-0.38** | -0.17 |
| 59 | Left Fusiform gyrus | **-20.2** | **-4.8** | -1.9 | -1.1 | **-0.32** | -0.20 |
| 60 | Right Fusiform gyrus | **-22.7** | **-6.3** | -3.0 | -1.6 | **-0.37** | -0.23 |
| 61 | Left Postcentral gyrus | **-14.1** | **-3.3** | **-7.7** | **-2.9** | **-0.30** | **-0.28** |
| 62 | Right Postcentral gyrus | **-15.5** | **-3.8** | -1.8 | -0.6 | **-0.28** | -0.22 |
| 63 | Left Superior Parietal gyrus | **-15.3** | **-2.4** | -0.2 | -0.1 | -0.22 | **-0.31** |
| 64 | Right Superior Parietal gyrus | **-17.7** | **-3.1** | -6.2 | -1.4 | **-0.25** | **-0.34** |
| 65 | Left Inferior Parietal gyrus | **-13.2** | **-3.0** | -5.8 | -1.9 | **-0.41** | **-0.32** |
| 66 | Right Inferior Parietal gyrus | **-12.4** | **-2.7** | -3.6 | -1.0 | **-0.32** | **-0.28** |
| 67 | Left SupraMarginal gyrus | **-13.3** | **-3.8** | **-13.6** | **-3.1** | **-0.26** | -0.19 |
| 68 | Right SupraMarginal gyrus | **-14.8** | **-4.0** | -3.7 | -0.7 | **-0.25** | **-0.28** |
| 69 | Left Angular gyrus | **-14.2** | **-3.5** | **-15.8** | **-3.3** | **-0.30** | -0.19 |
| 70 | Right Angular gyrus | **-17.2** | **-4.3** | -5.3 | -1.0 | **-0.41** | **-0.34** |
| 71 | Left Precuneus | **-18.4** | **-3.5** | -0.6 | -0.2 | **-0.42** | **-0.39** |
| 72 | Right Precuneus | **-19.6** | **-3.9** | -2.1 | -0.8 | **-0.35** | **-0.31** |
| 73 | Left Paracentral lobule | **-15.4** | **-3.0** | -1.4 | -0.3 | **-0.29** | -0.20 |
| 74 | Right Paracentral lobule | -5.9 | -1.0 | 4.3 | 1.0 | **-0.29** | **-0.32** |
| 75 | Left Caudate nucleus | **-12.4** | **-2.4** | -3.0 | -0.9 | **-0.30** | -0.18 |
| 76 | Right Caudate nucleus | **-17.6** | **-3.9** | -2.5 | -0.8 | **-0.25** | -0.24 |
| 77 | Left Putamen | **-10.6** | **-2.4** | **-12.4** | **-4.7** | **-0.26** | -0.18 |
| 78 | Right Putamen | **-10.7** | **-2.5** | **-14.3** | **-6.3** | -0.21 | **-0.26** |
| 79 | Left Pallidum | -2.7 | -0.6 | -1.2 | -0.4 | -0.15 | -0.18 |
| 80 | Right Pallidum | -6.6 | -1.5 | -0.6 | -0.2 | -0.13 | 0.01 |
| 81 | Left Thalamus | **-20.1** | **-3.5** | **-6.4** | **-4.6** | **-0.29** | -0.24 |
| 82 | Right Thalamus | **-20.7** | **-3.8** | **-7.8** | **-4.6** | -0.20 | **-0.32** |
| 83 | Left Heschl’s gyrus | **-15.2** | **-4.0** | -9.0 | -2.7 | -0.23 | -0.24 |
| 84 | Right Heschl’s gyrus | **-12.4** | **-2.9** | -6.5 | -1.9 | -0.20 | **-0.29** |
| 85 | Left Superior Temporal gyrus | **-12.0** | **-3.2** | **-12.5** | **-4.5** | **-0.29** | -0.19 |
| 86 | Right Superior Temporal gyrus | **-12.3** | **-3.7** | **-9.9** | **-4.1** | **-0.31** | **-0.33** |
| 87 | Left Temporal pole: Superior Temporal gyrus | **-15.0** | **-4.8** | 1.9 | 0.7 | **-0.32** | -0.19 |
| 88 | Right Temporal pole: Superior Temporal gyrus | **-15.4** | **-4.2** | 2.1 | 0.7 | **-0.38** | **-0.27** |
| 89 | Left Middle Temporal gyrus | **-12.3** | **-3.4** | -7.2 | -2.5 | **-0.41** | -0.24 |
| 90 | Right Middle Temporal gyrus | **-14.6** | **-3.9** | -4.4 | -1.7 | **-0.37** | **-0.30** |
| 91 | Left Temporal pole: Middle Temporal gyrus | **-18.3** | **-5.1** | 8.9 | 2.1 | **-0.33** | -0.17 |
| 92 | Right Temporal pole: Middle Temporal gyrus | **-15.3** | **-3.6** | 1.6 | 0.4 | **-0.35** | -0.18 |
| 93 | Left Inferior Temporal gyrus | **-20.3** | **-5.0** | -5.7 | -2.8 | **-0.30** | -0.02 |
| 94 | Right Inferior Temporal gyrus | **-20.4** | **-4.9** | -2.2 | -1.0 | **-0.34** | **-0.26** |
| 95 | Left Crus I of Cerebellar hemisphere | **-27.8** | **-6.4** | -5.2 | -2.0 | **-0.33** | -0.17 |
| 96 | Right Crus I of Cerebellar hemisphere | **-32.0** | **-8.2** | -7.9 | -2.1 | -0.22 | -0.23 |
| 97 | Left Crus II of Cerebellar hemisphere | **-21.2** | **-4.8** | -2.5 | -0.8 | -0.23 | -0.12 |
| 98 | Right Crus II of Cerebellar hemisphere | **-22.2** | **-5.4** | -3.7 | -1.2 | -0.23 | **-0.26** |
| 99 | Left Lobule III of Cerebellar hemisphere | **-15.7** | **-3.0** | 4.0 | 1.1 | **-0.26** | **-0.34** |
| 100 | Right Lobule III of Cerebellar hemisphere | **-15.5** | **-3.4** | -0.4 | -0.1 | -0.21 | -0.09 |
| 101 | Left Lobules IV-V of Cerebellar hemisphere | **-14.9** | **-4.4** | -5.3 | -1.8 | **-0.31** | **-0.29** |
| 102 | Right Lobules IV-V of Cerebellar hemisphere | **-18.1** | **-4.4** | -7.2 | -2.3 | **-0.33** | -0.23 |
| 103 | Left Lobule VI of Cerebellar hemisphere | **-18.0** | **-4.6** | -6.9 | -2.5 | **-0.30** | -0.25 |
| 104 | Right Lobule VI of Cerebellar hemisphere | **-21.3** | **-5.4** | -8.9 | -2.5 | **-0.30** | -0.21 |
| 105 | Left Lobule VII-B of Cerebellar hemisphere | **-19.3** | **-4.6** | 2.2 | 0.6 | **-0.35** | -0.14 |
| 106 | Right Lobule VII-B of Cerebellar hemisphere | **-19.8** | **-4.3** | -1.0 | -0.2 | **-0.28** | -0.11 |
| 107 | Left Lobule VIII of Cerebellar hemisphere | **-12.0** | **-2.4** | 5.1 | 1.8 | -0.23 | -0.09 |
| 108 | Right Lobule VIII of Cerebellar hemisphere | **-16.9** | **-3.8** | 3.6 | 1.3 | -0.25 | -0.22 |
| 109 | Left Lobule IX of Cerebellar hemisphere | **-17.2** | **-4.4** | -0.8 | -0.3 | -0.17 | -0.20 |
| 110 | Right Lobule IX of Cerebellar hemisphere | **-17.9** | **-4.7** | -2.8 | -1.2 | -0.23 | -0.22 |
| 111 | Left Lobule X of Cerebellar hemisphere | -6.9 | -1.1 | **14.3** | **4.9** | -0.22 | -0.23 |
| 112 | Right Lobule X of Cerebellar hemisphere | **-17.6** | **-2.7** | **19.4** | **4.7** | -0.18 | -0.23 |
| 113 | Lobules I-II of Vermis | - | - | 2.1 | 0.3 | **-** |  |
| 114 | Lobule III of Vermis | **-17.4** | **-3.5** | 17.2 | 4.5 | -0.20 | -0.21 |
| 115 | Lobules IV-V of Vermis | **-17.9** | **-4.3** | 0.3 | 0.1 | -0.23 | -0.25 |
| 116 | Lobule VI of Vermis | **-17.5** | **-3.1** | -1.8 | -0.6 | **-0.28** | **-0.28** |
| 117 | Lobule VII of Vermis | **-18.9** | **-2.9** | -5.3 | -1.3 | -0.21 | -0.20 |
| 118 | Lobule VIII of Vermis | **-22.0** | **-4.3** | -0.3 | -0.1 | -0.21 | **-0.26** |
| 119 | Lobule IX of Vermis | **-22.4** | **-5.5** | -4.3 | -0.9 | **-0.25** | **-0.32** |
| 120 | Lobule X of Vermis | **-20.9** | **-3.9** | 9.6 | 1.9 | -0.24 | **-0.33** |
